# Suffering Makes You Egoist: Acute Pain Increases Acceptance Rates and Reduces Fairness during a Bilateral Ultimatum Game

**DOI:** 10.1371/journal.pone.0026008

**Published:** 2011-10-12

**Authors:** Alessandra Mancini, Viviana Betti, Maria Serena Panasiti, Enea Francesco Pavone, Salvatore Maria Aglioti

**Affiliations:** 1 Psychology Department, Sapienza, University of Rome, Rome, Italy; 2 Istituto di Ricovero e Cura a Carattere Scientifico (IRCCS), Santa Lucia Foundation, Rome, Italy; 3 Institute for Advanced Biomedical Technologies, G. d'Annunzio, Univeristy of Chieti, Chieti, Italy; French National Centre for Scientific Research, France

## Abstract

Social preferences like interpersonal altruism, fairness, reciprocity and inequity aversion are inherently linked to departures from pure self-interest. During economic interactions, for example, defectors may be punished even if this implies a cost for the punishers. This violation of canonical assumptions in economics indicates that socially oriented decisions may predominate over self-centred stances. Here we explore whether the personal experience of pain changes the balance between self-gain and socially based choices. We used laser stimulation to induce pain or a warm sensation in subjects playing a modified version of the Ultimatum Game (UG) both in the role of responder and proposer. After each shot, responders evaluated the fairness of the offer. Moreover, responders and proposers rated the intensity and unpleasantness of the sensation evoked by the laser stimulation. Results show that suffering proposers decrease fair offers and suffering responders increase their acceptance rate irrespective of economic offer. Crucially, the intensity of painful stimulation has a predictive role on Moderately Unfair offers' acceptance rates. Thus the personal experience of pain may favour the emergence of a self-centered perspective aimed at maximizing self-gain. The results suggest that bodily states play a fundamental role in higher-order interpersonal negotiations and interactions.

## Introduction

Canonical models of economics posit that material self-interest is the sole motivational force guiding human behavior. This picture is challenged by the observation that under various circumstances people take into account also the welfare of others [1]. Modern economics and psychological theories [2], [3], [4] support the notion that altruistic, fair and trusting behaviors represent essential ingredients of strategic interactions. The view of human nature as not purely self-regarding but also including *social preferences* (e.g. interpersonal altruism, fairness, reciprocity and inequity aversion) inspired combined neuroimaging techniques and game theory studies, aimed at exploring the brain processes underlying the implementation of specific social behaviors like fairness [5]. A routinely employed task is the Ultimatum Game (UG) [1], where one player (proposer) plays as the first mover and decides how to divide a given amount of money (e.g. € 10) in an anonymous *one-shot* interaction. In this condition, negotiation effects are ruled out by the absence of repeated plays. The proposer decides how to split the stake with the only constraint that the responder cannot get 0 (e.g. € 8 for him/her, € 2 for the other player). If the responder accepts, each player keeps the allocated amount of money; if he/she rejects the offer, both players receive nothing. According to standard economic models, in order to maximize his/her own payoff the responder should accept any offer. Indeed, although inequitable, any offer is better than nothing. However, in accordance with theories of reciprocity [6] and inequity aversion [2], participants systematically reject unfair offers below the 20–30% of the total pot [7], [4], preferring to gain nothing rather than accept unequal distribution of resources [5]. The human tendency to punish defectors has a vital role in maintaining cooperation and converting individual loss into a gain for the group, even though it yields no direct benefits or is even costly to the punisher [8], [9]. Experimental economic games provide a unique opportunity to elicit social preferences and measure how much players are willing to sacrifice their own economic payoff if this allows them to punish defectors [4], [10]. Indeed in the course of the last decade, researchers attempted to understand the brain processes that govern social preferences like fairness, using modern neuroimaging techniques. Pioneer fMRI [11], [12] and TMS studies [13], [14] using the ultimatum game (UG), for example, hint at the existence of a neuronal circuitry comprising the dorsolateral prefrontal cortex, the bilateral anterior insula and the anterior cingulate cortex specifically involved in the perceived fairness of others [11].

Studies on the objective and contextual aspects of fairness highlight the different neural underpinnings of objective social inequality with respect to contextual aspect of fairness [15]. Moreover, behavioral studies show that incidental negative emotion influences behavioral responses [16], [17], [18] and fairness ratings [16]. Using an iterated version of *one-shot* UG, it has been shown, for example that emotion of disgust elicits higher rejection rate of unfair offers, with respect to sadness and neutral states [16]. Thus, emotions may shape social preferences, and in some circumstances exacerbate the tendency to punish free-riders.

Little is known about whether the personal experience of pain modulates the rejection of unfair offers or the decision to reciprocate altruistically. Although pain has been considered as an inherently private experience, recent neuroscientific evidence indicates that the first-hand experience of pain makes individuals more prone to react to the pain of others according to egocentric rather than to other-oriented stances, adopting a less empathic attitude. [19].

Here, we expand current knowledge by attempting to determine whether acute pain reduces social preferences as indexed by an iterated version of the *one-shot* UG. We employed a bilateral version of the ultimatum game where participants alternatively acted as proposer or responder while receiving on the dorsum of the left hand, laser stimuli that could induce acute pain (Pain condition) or a warm sensation (Heat condition). The procedure allowed us to explore whether being in pain specifically affects the decision of a given individual to accept a given amount of money when playing in the role of responder and the way in which he/she divides a sum of money when playing in the role of proposer. Finding higher acceptance rates of unfair offers and lower offers in the pain with respect to heat condition would support the notion that pain perception may induce a self-centered bias that ultimately inhibit the tendency to implement socially oriented behaviors like altruistic punishment.

## Materials and Methods

### Participants

Thirty healthy right-handed subjects (14 female; age range: 18 to 36 years (M = 23.93, SD = 4.56) recruited via an opportunity sample, participated in the study. Subjects were paid a fixed amount of 15 Euros. In addition, they were informed they would effectively receive the money earned during the economic game.

Participants gave their written consent and were naïve as to the purposes of the study. The experimental protocol was approved by the local Ethics Committee at the Fondazione Santa Lucia and the study was conducted in accordance with the ethical standards of the 1964 Declaration of Helsinki.

### The Ultimatum game

Subjects were told they were going to play an economic game against other participants located at other two remote Italian Universities, using an-internet based platform. They were also told that depending on the platform's requirement they were to play in different blocks the role of proposer or of responder. The proposer's role was to decide how to split 1 € while the responder's role was to decide whether to accept or reject the offer. If the responder accepted, each player would keep the allocated amount. If he/she rejected the offer, both players would receive nothing.

To rule out the possibility of any negotiation between participants, subjects were ensured that for each match they would be randomly paired with an anonymous partner. Unknown to the participants, the game took place against a PC device, which was programmed using E-Prime software 1.2.

### Laser stimulation

Laser stimulation was delivered with an infrared neodymium yttrium aluminium perovskite laser (EL.EN. Group) to the dorsum of the left hand. The laser stimulation allowed us to induce acute painful and warm sensation on the body part selectively stimulated by the laser beam without the concurrent experience of touch. We determined the individual heat and pain threshold according to the method of limits [20]. The threshold values corresponded to the lowest painful or warm sensation that can be reliably detected in 5 out of 10 trials and were determined before each experimental condition. Moreover, pain and heat thresholds were determined at the beginning of each block. The fluency of the stimuli used in the Pain and Heat Condition was 30% over the painful and warm threshold values; both in the responder (Pain = 14.8 J/cm^2^, ±4.0; Heat = 8.7 cm^2^±2.9), and in the proposer (Pain = 15.1 cm^2^, ±3.6; Heat = 9.2 cm^2^±2.1) role.

Laser pulses were delivered in blocks of 10 trials. To avoid nociceptors fatigue or sensitization, the location of the laser on the skin was slightly shifted after each stimulus. An area of about 8 cm^2^ on the radial side of the hand dorsum was stimulated. Moreover, a 5–7 seconds interstimulus interval (ISI) allowed us to minimize central habituation effects. The distance between the laser stimulator and the hand was kept constant (and was about 2 cm).

### Experimental Procedure

**Figure 1:**
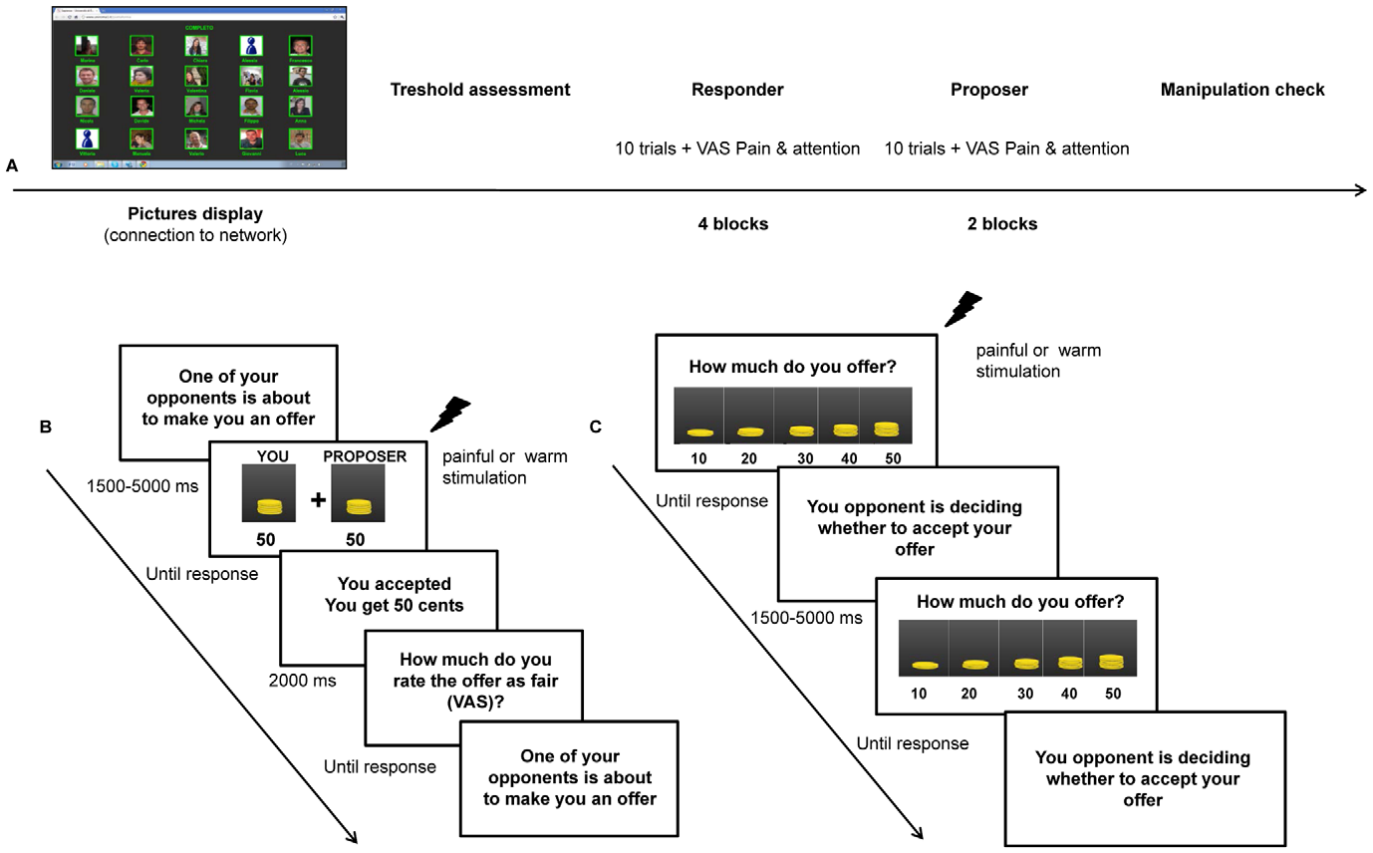
Bilateral Ultimatum Game procedure. Panel A schematically represents the entire procedure. Subjects were introduced to the internet-based platform which displayed the pictures of the confederates. After the determination of the laser threshold, participants played first as responders (four blocks of 10 trials each) and then as proposers (two blocks of 10 trials each). Finally, they were presented with a manipulation check. Panel B: represents the sequence a typical event trial of the responder's blocks. After a variable interval, subjects received the offer contemporaneously to a painful or warm laser stimulation. They could accept or reject the offer by means of a button press. Subsequently, a feedback informed participants how much they received and they could rate the fairness of the offer on a VAS scale ranging from 0 (unfair) to 100 (fair). Panel C represents the sequence of events in a trial of the proposer's blocks. Participants had to decide how to split money (1€), selecting the corresponding offer by clicking with the cursor on the image displayed on the screen. No feedback was provided in order to avoid that the responders' choice influences the subsequent offer.

Subjects were seated in a comfortable armchair and were asked to relax their muscles but to stay alert. In order to avoid that they could explicitly realize we wanted to measure the influence of pain on their social preferences, participants were told that the aim of the study was to assess their subjective pain threshold while they were committed in a distracting game (the UG). Figure 1 schematically represents the procedure. Each subject was preliminarily introduced to the internet-based platform in order to familiarize with the procedure and to visualize the faces of the confederates who gave their written informed consent (as outlined in the PLoS consent form) to publication of their pictures.

After the determination of the laser pain and heat thresholds, specific instructions prompted subjects to play the responder or the proposer role (figure 1A). In the *responder* blocks, subjects were asked to accept of refuse the offer of other confederates, as follows (translated from Italian): “The computer randomly assigned you the role of responder. You may accept or reject the offers that come from your opponents. If you accept, the money will be divided according to the offer, if you reject neither of you will receive nothing”. In the *proposer* blocks subjects were instructed to decide how to divide the sum of money, as follows (translated from Italian): “The computer randomly assigned you to the role of proposer. You may decide how to allocate the money. If your opponent accepts the offer, the money will be divided accordingly, if he/she rejects the offer, no money will be given to any of you”. In the *responder* blocks subjects accepted or rejected the offer by pressing a button (left to accept, right to reject) with their right hand. At the end of each interaction, a feedback lasting 4 seconds informed participants about how much each player received (for example, “you get € 30 cents” or “you get € 0” if the offer was accepted or rejected, respectively) (figure 1B).

In the *proposer* blocks, participants had to decide how to split money by clicking on one of five possible offers displayed on the screen (figure 1C). In these blocks no feedback was provided to avoid any effect of the outcome on the subsequent offer.

Overall, each subject was tested in six experimental blocks. In the first four blocks, participants were assigned to the responder role (*responder* blocks), while in the remaining two blocks they played as proposers (*proposer* blocks). For each subject, the *responder* block was repeated twice, one for Pain and one for Heat Condition. This procedure ensured an adequate amount of iterations. On each block, participants completed 10 trials, for an overall amount of 60 iterations in the whole experiment. The experiment lasted 1 hour. Laser pulses were delivered at the onset of each trial. The order of Heat and Pain Condition was counterbalanced across subjects.

Both in the *responder* and in the *proposer* blocks, possible offers ranged from 50 cents to 10 cents, as follows: 50 cents (Fair, *F*), 40 cents (Moderately Fair, *MF*), 30 cents (Moderately Unfair, *MU*), 20 (Unfair, *U*) and 10 cents (Extremely Unfair, EU). Each responder block included 10 offers according to the following: 3×50 cents, 2×40 cents, 3×30 cents, 1×20 cents and 1×10 cents. *Fair* offers were restricted to 50 cents assuming that confederates would not offer more than half of the amount. U and EU offers were limited since are routinely rejected (4). At the end of the experiment subjects were debriefed about the purpose of the study.

### Subjective ratings of offers' fairness and laser stimuli

After each trial, participants were asked to assess the fairness of each offer on a Visual Analogue Scale (VAS) ranging from 0 (unfair) to 100 (fair). The question (translated from Italian) was the following: “on a scale of 0 to 100, where 0 corresponds to unfair and 100 to fair, how would you rate the offer you have just received?”. Furthermore, at the end of each block participants were asked to rate the intensity and the unpleasantness of the laser stimulation, along a VAS where 0 corresponded to no pain (intensity or unpleasantness) and 100 the maximum pain that can be imagined. Finally, subjects were instructed to maintain attention to the stimuli and to monitor the interaction. This allowed to check for unwanted fluctuations of attention in the different blocks. Moreover, participants evaluated the attention they addressed to the task and to the stimulation, to assess whether they varied across blocks.

### Manipulation check

Immediately after the experiment, participants completed a four-items questionnaire investigating their feelings about the experimental task. In particular, they were asked the following questions (translated from Italian): 1) how much did you use a pre-defined strategy during the UG (e.g. you decided a-priori to accept any offer above 30 cents), 2) how much did you feel angry at your opponents, 3) how much did you feel prone to accept, 4) did you feel involved in the interaction with your opponents even if you could not see their faces? For item 1 to 3, evaluations along a 5-point Likert scale (ranging from −2 to +2) were required. Separate questions for the Pain and Heat conditions were asked. For item 4 a mere “yes” or “no” response was contemplated. Six subjects who declared the lack of involvement in the interaction with the other players or spontaneously expressed scepticism about the real existence of the confederates were excluded from the analysis.

## Results

### Data handling

Data analysis was performed on 24 subjects (12 females; age range 18–36 M = 23.92, SD = 4.75). In the *responder* blocks, we obtained the acceptance rate (%) for each subject dividing the frequency of the accepted offers for Fair (or Unfair) by the total of number of Fair (or Unfair) items. In the *proposer* blocks, we computed the offer rate (in %) for each subject. In details, the frequency of each offer type (10, 20, 30, 40, 50 cents) was expressed as percentage of the total number of items within each block. Due to unexpected interruption of the experiment caused by technical problems, two subjects could not finish the proposer trials and were excluded from the analysis performed on offer rate.

In the responder blocks, acceptance rates of 40 and 50 offers and of 30, 20 and 10 offers were collapsed in Fair and Unfair categories respectively. This procedure allowed us to compare the same number of trials for each category.

Values of acceptance rate (%) were analyzed by means of a 2×2 repeated-measures ANOVA with Condition (two levels: Pain and Heat) and Fairness of Offer (two levels: *Fair and Unfair*) as main factors. The same analysis was performed on VAS ratings of Fairness and reaction times (RTs).

In the *proposer* blocks a 2×5 repeated-measures ANOVA was performed on offer rate (%) values with Condition (two levels: Pain and Heat) and Fairness of Offer (five levels: *10*, *20*, *30*, *40*, *50 cents*) as main factors. A dependent sample t-test was performed to check for any difference in the RTs of the two conditions. Moreover, we performed standard multiple regression models on subjective ratings of intensity and unpleasantness of the painful laser stimulation (as independent variables) and the acceptance rate of each offer in the responder block and the frequency of each type of offer in the proposer block (as dependent variables). Finally, we performed four separate 2×2 repeated-measures ANOVAs on i) Intensity and ii) Unpleasantness of laser stimulation, iii) attention to stimulation and iv) attention to the task, with Role (two levels, *responder* and *proposer*) and Condition (Pain and Heat) as main factors. *Post-hoc* comparisons were performed by means of Newman-Keuls test.

### Acceptance rates in the responder blocks

**Figure 2:**
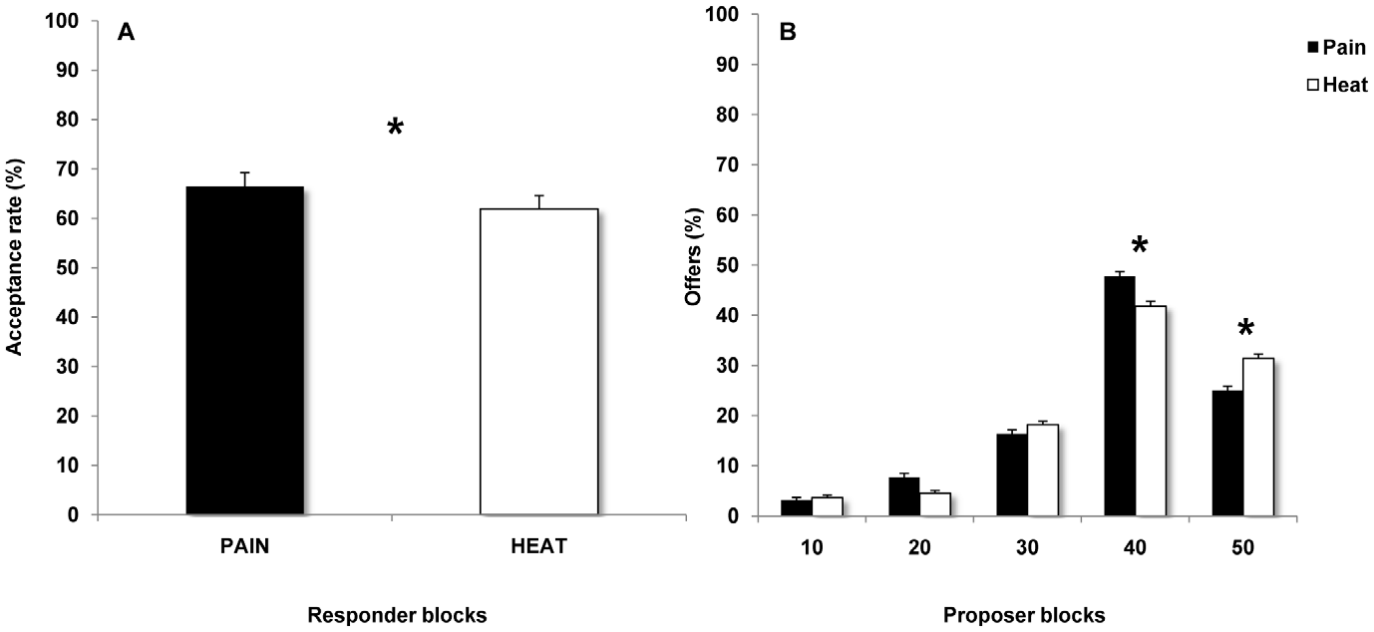
Pain induces self-regarding preferences. Panel A shows the higher acceptance rate observed in the Pain with respect to Heat condition in the responder's blocks. Panel B shows the significant interaction Fairness of Offer×Condition, accounted for by a higher rate of Moderately Fair offers (40 cents) in Pain with respect to Heat Condition and a lower rate of Fair offers (50 cents) in Pain with respect to Heat Condition in the proposer's blocks.

In the *responder* blocks, participants modulated their acceptance rate as a function of Condition as explained by the significance of the main effect (*F_1,23_ = 7.20*, *p = 0.013*, *η^2^_p_ = 0.23*). Results showed a higher acceptance rate during Pain with respect to Heat (Figure 2A). As expected, the acceptance rate was also higher for *Fair* with respect to *Unfair* offers, as revealed by the significance of the main effect (*F_1,23_ = 170.40*, *p<0.000*, *η^2^_p_ = 0.88*).

The interaction Condition×Offer was not significant (*F_1,23_ = 0.42*, *p = 0.52*). Since acceptance of fair offers is usually at ceiling in the UG, this lack of significance may depend on the way in which data were collapsed. Thus, we run an additional ANOVA considering four levels of Fairness of Offer, namely: Fair (50 cents), Moderately Fair (40 cents), Moderately Unfair (30 cents) and Unfair (10–20 cents) as main factors. The results were identical to those obtained running a 2×2 ANOVA. Indeed, we found significant main effects of Condition (*F_1,23_ = 5.35*, *p = .03*, *η^2^_p_ = .19*), and of Fairness of Offer (*F_3,69_ = 109.63*, *p<.0000*, *η^2^_p_ = .83*) but no significant Condition×Offer interaction (*F_3,69_ = 1. 48*, *p = 0.23*, *NS*). Importantly, Newman-Keuls *post-hoc* comparisons revealed that participants accepted Fair and Moderately Fair offers at a similar rate (*p = .45*, *NS*) (acceptance rates raw data % are reported in Table 1).

**Table 1:** Acceptance rate raw data (%).

	Acceptance Rate Pain (%)				Acceptance Rate Heat (%)			
Subjects	Unfair	Moderately Unfair	Moderately Fair	Fair	Unfair	Moderately Unfair	Moderately Fair	Fair
**1**	0	83,33	100	100	0	66,7	100	100
**2**	0	100	100	100	0	100	100	100
**3**	0	100	100	100	0	100	100	100
**4**	25	50	100	100	0	34	100	100
**5**	0	0	100	100	25	0	75	100
**6**	0	100	100	100	0	50	100	100
**7**	0	16,6	100	100	0	33,3	100	100
**8**	0	50	100	100	25	33,4	100	100
**9**	0	16,67	75	100	0	0	100	100
**10**	0	0	100	100	0	0	100	100
**11**	25	16,67	100	100	25	0	100	100
**12**	0	66,67	100	100	0	0	75	100
**13**	25	100	100	100	0	83,34	75	100
**14**	50	100	100	100	75	100	100	83,34
**15**	0	100	100	100	0	100	100	100
**16**	25	83,34	100	100	75	83,34	100	100
**17**	25	33,34	100	100	0	16,67	75	100
**18**	0	66,67	75	83,34	25	0	50	100
**19**	25	0	100	100	0	16,67	100	100
**20**	25	100	100	100	0	100	100	100
**21**	0	33,34	100	100	0	50	75	100
**22**	0	0	100	100	0	0	75	100
**23**	0	0	100	100	0	16,67	100	83,34
**24**	0	16,67	75	100	0	33,4	100	83,34
**MEAN**	**9,37**	**51,39**	**96,9**	**99,3**	**10,41**	**42,39**	**91,66**	**97,91**
**SD**	**14,39**	**40,5**	**8,44**	**3,4**	**22,01**	**39,3**	**14,11**	**5,63**
**SE**	**0,16**	**0,26**	**0,12**	**0,07**	**0,19**	**0,26**	**0,15**	**0,09**

The table reports the acceptance rate (%) of Unfair (10–20 cents), Moderately Unfair (30 cents), Moderately Fair (40 cents) and Fair (50 cents) offers presented by each participant in the Pain (left column) and in the Heat (right column) condition.

**Figure 3:**
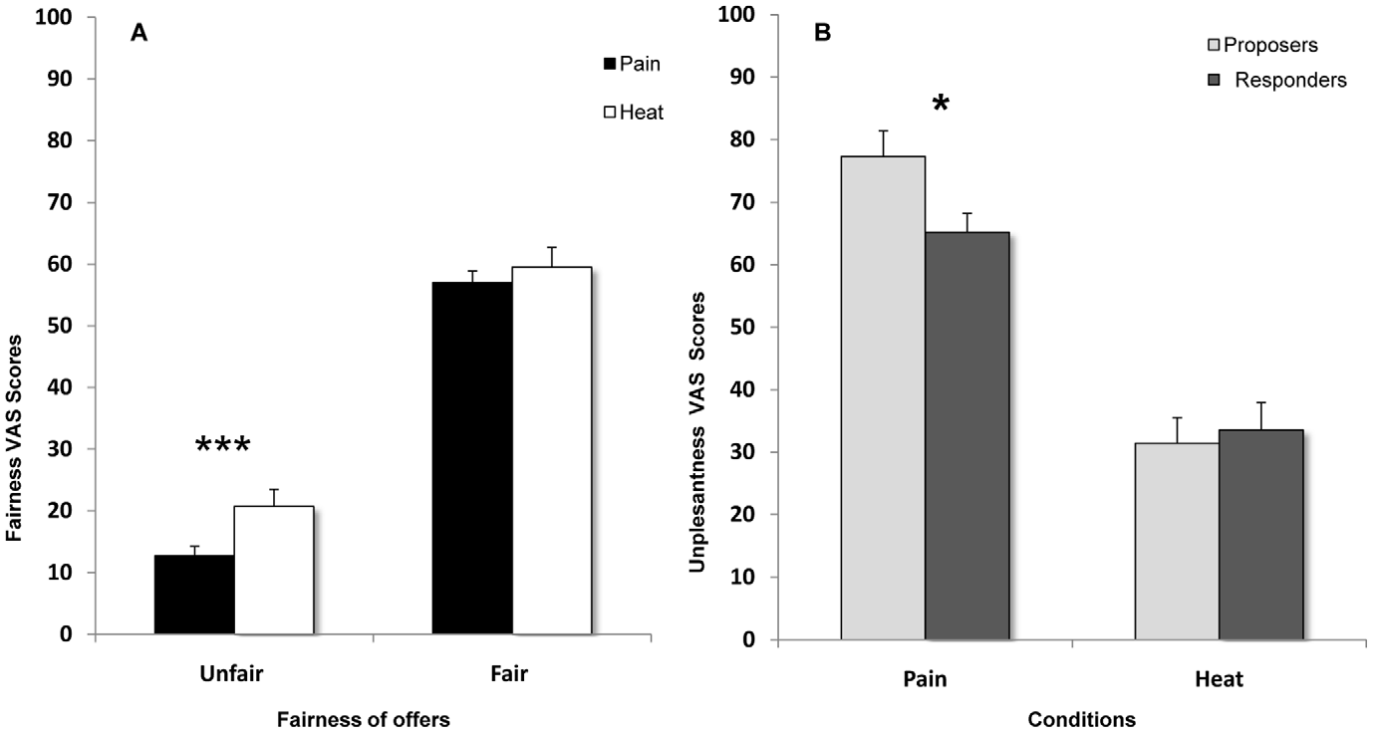
Fairness and Pain ratings. Panel A shows a significant interaction between Fairness of Offer and Condition, revealing that subjects expressed more severe judgments for unfair offers in the Pain Condition with respect to the Heat Condition during the responder's blocks. Panel B shows the significant interaction between Condition and Role, revealing that subjects judged more unpleasant the painful laser stimulation while acting as proposers than as responders.

*Fairness scores.* ANOVA performed on Fairness scores revealed higher scores for *Fair o*ffers with respect to *Unfair* offers, as shown by the main effect of Fairness of Offer (*F_1,23_ = 129.12*, *p<0.000*, *η^2^_p_ = 0.85*). Importantly, fairness scores were lower in Pain with respect to Heat condition, as indicated by the main effect of Condition (*F_1,23_ = 39.98 p<0.000 η^2^_p_ = 0.63*). Crucially, we found a significant interaction Condition×Fairness of Offer (*F_1,23_ = 6.99 p = 0.014 η^2^_p_ = 0.23*) which was entirely accounted for by lower VAS scores for Unfair offers during Pain with respect to the Heat condition (*p<0.001*, Newman Keuls *post-hoc*) (Figure 3A).

*Reaction Times.* ANOVA performed on RTs revealed a main effect of Fairness of Offer (*F_1,23_ = 21.80*, *p<0.001*, *η^2^_p_ = 0.49*), explained by higher RTs to Unfair offers than to Fair offers (*p<0.001*). The main effect of Condition and its interaction with the Fairness of Offer did not reach the significance (all *ps>0.05*).

### Intensity of painful stimulation was predictive of acceptance rates for moderately unfair offers

The only significant regression model was for 30 cents offers (*R = 0.55*, *Adj R^2^ = .23*, *F = 4.51*, *p<.05*). In particular, for this type of offer the intensity of the painful stimulation was predictive of the acceptance rates (*ß = 0.61*, *t_21_ = 2.92*, *p<0.01*). For regression analyses, we computed the Cohen's *f* 2: R^2^/(1−R^2^) as an index of effect size. Cohen's *f* 2 was computed on the AdjR^2^ (*f*2 = .29). Both Intensity and Unpleasantness of the warm stimulation were not predictive of subject's acceptance rates in the Heat condition.

### Offering behaviour in the proposer blocks

We found a significant main effect of the Fairness of Offer (*F_4, 84_ = 24.5*, *p<0.000*, *η^2^_p_ = 0.54*) which was entirely accounted for by higher percentage of *MF* (40 cents) with respect to *F* (50 cents) offers (p<0.01) and *U* offers (all *Ps<0.001*). Crucially, we found a significant interaction between Fairness of Offer and Condition (*F_4, 84_ = 2.9*, *p = 0.026*, *η^2^_p_ = 0.12*). Specifically, we observed a higher percentages of *MF* (40 cents) offers in Pain with respect to Heat Condition (*p = 0.035*) and lower percentages of Fair offers (50 cents) in Pain, with respect to Heat Condition (*p = 0.024*) (Figure 2B). The main effect of Condition did not reach the significance (*F_1, 21_ = 1.0*, *p = 0.33*).

The regression model performed on proposer blocks was not significant (all *Ps>.05*).

Reaction times were not significantly different between conditions (*t = .33*, *p = .75*, *NS*).

#### Intensity and Unpleasantness Ratings

Subjective measures showed a significant effect of Condition both for Intensity (*F_1,21_ = 153.36*, *p<0.000*) and Unpleasantness scores (*F_1,21_ = 73.58*, *p<0.000*). *Post-hoc* test revealed higher VAS scores for Pain with respect to Heat (all *Ps<0.000*). We did not find a significant effect of Role on laser pain Intensity (*F_1,21_ = 0.08*, *p = 0.78*) or Unpleasantness (*F_1,21_ = 2.04*, *p = 0.17*). Importantly a significant interaction Role×Condition (*F_1,21_ = 4.88*, *p = 0.038*) was found only for laser pain Unpleasantness. *Post-hoc* tests showed that in the Pain condition, unpleasantness scores were higher when subjects played as proposers with respect to when they played as responders (*p = 0.015*) (Figure 3b). No such difference was found in the Heat Condition (*p = 0.64*, *p>0.05*).

#### Attention VAS scores

ANOVA performed on subjective ratings of attention to stimulation revealed a significant main effect of Condition (*F_1,21_ = 63.54*, *p<0.000*) showing higher VAS scores during Pain with respect to Heat Condition (*p<0.000*). Neither Role nor its interaction with Condition were significant (all *Ps>0.05*). Subjective ratings of the amount of attention that participants devoted to the UG task revealed a lack of significance both for Condition (*F_1,21_ = 2.23*, *p = .15*) and Role (*F_1,21_ = 1.91*, *p = 0.19*) as main factors, as well as for their interaction (*F_1,21_ = 1.91*, *p<.18*).

### Manipulation check

Subjects reported higher ratings of anger when they were in the Pain condition with respect to Heat (*t = 3.71*, *p<0.001*) and reported to feel themselves more prone to accept when they were in the Heat condition with respect to Pain condition (*t = −2.12*, *p<0.04*). Interestingly, such subjective impression contrasts with the actual behaviour of the participants who accepted more offers in the Pain condition. Scores related to the use of an a-priori strategy (e.g. accepting any offer below a given value) were not statistically significant between the two conditions (*t = −.97*, *p = .34*, *NS*).

## Discussion

Recent behavioral studies highlighted the crucial role of incidental negative emotions, as disgust [16], sadness, and anger [17], [18] in exacerbating the human tendency to punish defectors, an index of prosocial behavior [1]. Here, using a bilateral iterated version of *one-shot* UG, we demonstrate that first-hand experience of pain strongly modulates the strategic economic interaction in participants playing either the responder or the proposer role. In particular, we show that feeling pain makes an individual less inclined to behave according to the social norms (e.g. punishment of defectors) that regulate most social and economic interactions.

### Pain triggers a self-centred perspective when playing the UG as responder

A plausible interpretation of the fact that people generally prefer to behave altruistically is that subjects derive higher hedonic value from the mutual cooperation outcome [21], [22], [12]. Consistently with this interpretation, it is widely held that the brain uses a common-reward metric for the processing of both individual and social rewards [23]. Interestingly, there is evidence that fairness-directed conducts, such as mutual cooperation with a human partner [24], donating to a charity [25], [26], altruistic punishment and revenge [27], [28] are related to neural activation of the mesolimbic dopaminergic system. We sought to determine whether urgent and unpleasant framing, such as that elicited by an acute painful stimulation, may shift people's preference towards the individual reward (i.e. monetary gain). Our results show that perceiving pain specifically elicits higher acceptance rates in subjects playing as responders in a bilateral iterated version of *one-shot* UG. No such effect was induced by non-noxious heat stimuli. This result expands previous findings revealing that the personal experience of pain influences social interactions by inducing an egocentric bias and reducing the capacity to react empathically toward others [19]. It is worth noting that the acceptance rates were higher in the Pain condition irrespectively of the fairness of the offer, suggesting that the perception of pain favours the emergence of a maximizing behavior. Such behavior allows subjects to make choices aimed at achieving the highest possible gain. Interestingly, this effect is reminiscent of what found in chronic back pain (CBP) patients playing the Iowa Gambling Task [29], a card game developed to study emotional decision-making [30]. Notably, CBP patients tended to choose more frequently cards from the bad decks (those that yielded high immediate gain but larger future losses) with respect to control subjects. Furthermore, the performance of these patients turned out to be associated with the intensity of chronic pain [29]. We showed that the intensity of the painful stimulation is predictive of the higher acceptance rate of moderately unfair offers (MU) (i.e. 30 cents) that correspond to 30% of the total pot. Interestingly this is the percentage at which altruistic punishment starts to occur [4]. Thus, our results raise the possibility that perceiving pain strongly influences the economic interaction, inducing suffering individuals to behave according to selfish motives. It is worth noting that the enhancement of acceptance rate found in the Pain condition cannot be attributed to a decreased moral standard in our participants. On the contrary, participants were more severe, assigning lower scores to unfair offers in the Pain with respect to Heat condition. A similar dissociation between appraisal and actual behavior was found in a low-frequency repetitive transcranial magnetic stimulation (rTMS) study [13]. rTMS inhibition of the right dorsolateral prefrontal cortex reduced the subjects' ability to resist to the selfish temptation to accept intentionally unfair offers, but preserved the ability to judge low offers as unfair [13]. Additional evidence for this segregation comes from a clinical study [31] which examines the economic behaviour of patients with focal lesions of ventromedial prefrontal cortex in comparison to that of patients with damage sparing the frontal cortex and of healthy subjects. Confirming previous evidence [32], the results showed that, when playing the standard version of the UG, patients with lesions to the ventromedial prefrontal cortex rejected unfair offers at higher rate than non-frontal patients and healthy subjects. Importantly, the lesion did not affect the judgment of unfair offers [31].

### Pain triggers a self-centred perspective when playing the UG as proposer

The behavior expressed by subjects acting in the proposer's role has been less explored in literature. To the best of our knowledge there is only one study which explored proposers' preferences and conducts. The study shows that sophisticatedly selfish proposers derived greater pleasure from payoffs patently unbalanced in their favor rather than from fair payoffs [33]. Consistently, we observed that when our subjects were playing as proposers their behavior appeared more strategic and less fair in the Pain than in the Heat condition. In the Pain but not in the Heat condition, participants offered more moderately fair (40 cents) than truly fair amounts (50 cents). This result complements and expands a recent study on the link between pain and money [34] that shows handling money may reduce pain sensitivity and that thinking of having spent money exacerbates physical pain.

The unpleasantness of the laser pain was rated as significantly more unpleasant when subjects played in the proposer than in the responder role. Although further investigation on this effect is needed, it hints at the complex interaction between bodily states and the role during economic interactions.

Participants reported they paid more attention to painful than warm stimuli. Thus, the higher acceptance rate and the decreased level of fairness reported by subjects during Pain conditions of the responder and proposer blocks, could depend on a lower amount of cognitive resources devoted to the UG task in Pain than in Heat conditions. However, the subjective ratings of the amount of attention that subjects devoted to the UG task, were comparable in Pain and Heat conditions. Moreover, the analysis performed on Reaction Times both in the Responder' and in the Proposer's role did not show a significant main effect of Condition. Taken together our results suggest that the cognitive resources allotted to the UG task were comparable in Pain and Heat conditions.

### Pain modulates interactive behavior differently from other negative emotions

Most of the research attempting to disentangle the role of negative emotions in the rejection of unfair offers has been conducted inducing the negative emotion before playing the game [16], [17], [18]. Interestingly, subjects who played the UG in the presence or absence of a disgusting odor showed a higher acceptance in the latter than the former context [35]. This effect seems to be gender-selective. Indeed, male participants reported higher disgust and judged the offer as less unfair than females. One plausible explanation for this result posits a spontaneous affective discounting where spontaneous misattribution of the disgust is typically associated to the unfair offer and to the disgusting environmental smell [35].

The manipulation check indicates that our subjects were more angry and less prone to accept in the Pain than in the Heat condition. It is worth noting that in our study painful stimulation and the offers perception were contemporary. In principle, participants might have misattributed the anger they felt for the unfair offer to the painful stimulation that they were receiving. This explanation seems unlikely for at least two reasons. First, the misattribution hypothesis [36], [35] is based on the appraisal theory that suggests specific cognitions are important antecedents of specific emotions and thereby of specific action tendencies [37], [38]. Were this the case, our participants should have presented higher fairness ratings for unfair offers in the Pain condition. As a matter of fact, subjects reported lower fairness ratings for the unfair offers in the Pain condition which is exactly the opposite pattern of results. On the contrary, they accepted more in the Pain condition irrespectively of the fairness of the offer.

In conclusion, some negative emotions like induced disgust, modulate social preferences differently from pain. This may be surprising because the above negative emotions are underpinned by neural regions, e.g. the insular cortex, that also represent pain [39], [40]. Thus, an additional point of interest of our paradigm is that it may be useful for investigating the neural correlates of induced social preferences.
