# Correction: Suffering Makes You Egoist: Acute Pain Increases Acceptance Rates and Reduces Fairness during a Bilateral Ultimatum Game

**DOI:** 10.1371/annotation/7bfc7dcf-e5f7-4222-9bf6-92aee46c5c09

**Published:** 2011-11-01

**Authors:** Alessandra Mancini, Viviana Betti, Maria Serena Panasiti, Enea Francesco Pavone, Salvatore Maria Aglioti

The authors wish to add the following statement to their Funding section: "Funded by the EU Information and Communication Technologies Grant (VERE project, FP7-ICT-2009-5, Prot. Num. 257695) and the Ministero Istruzione Università e Ricerca (Progetti di Ricerca di Interesse Nazionale, PRIN 2009)." 

